# Cetuximab-Induced Pneumonitis: An Overlooked Complication

**DOI:** 10.7759/cureus.72797

**Published:** 2024-10-31

**Authors:** Oluwatosin Emehinola, Ruhma Ali, Gunwant Guron, Richard Miller

**Affiliations:** 1 Internal Medicine, New York Medical College (NYMC) at St. Michael's Medical Center, Newark, USA; 2 Internal Medicine, St. Michael's Medical Center, Newark, USA; 3 Hematology and Oncology, St. Michael's Medical Center, Newark, USA; 4 Pulmonary and Critical Care Medicine, St. Michael's Medical Center, Newark, USA

**Keywords:** anti cancer drugs, anti-egfr, colorectal cancer (crc), drug-induced interstitial pneumonitis, drug induced lung injury, interstitial pneumonitis

## Abstract

Epidermal growth factor receptor (EGFR) inhibitors have largely been used for head and neck cancers, non-small cell lung cancers, and colorectal cancers (CRC). Reports of interstitial pneumonitis with EGFR inhibitors like gefitinib and erlotinib are present in the literature, but pulmonary toxicity with cetuximab has rarely been reported.

We present a case of a 78-year-old male with metastatic CRC involving the brain and lungs who presented with severe pneumonitis, a month after treatment with cetuximab. The patient was started on a chemotherapy regimen of 5-fluorouracil, leucovorin calcium, and irinotecan, and later began immunotherapy with cetuximab. Upon admission, the patient complained of shortness of breath. The CTA showed multifocal bilateral ground-glass opacities. Tests for influenza A and B RT-PCR were negative. Tests for Legionella antigen, strongyloides IgG antibody, histoplasma antibody/antigen, cryptococcal antigen, and HIV were also negative. The serum Fungitell assay was within normal limits. The QuantiFERON Gold Plus test was indeterminate, and the COVID-19 PCR was negative twice. The sputum culture was normal. Bronchoscopy could not be performed due to increased oxygen requirements. After excluding other differential diagnoses, cetuximab-induced pneumonitis was considered. The patient was given high-dose corticosteroid therapy; however, he continued to deteriorate clinically and expired after two weeks of hospital admission.

This case emphasizes the need for physician alertness in diagnosing drug-induced lung injury and suggests that other alternative disorders should not be ignored.

## Introduction

The use of precision oncology therapies for carcinomas has become increasingly prevalent to improve morbidity and mortality [1]. Cetuximab is a monoclonal antibody that targets the epidermal growth factor receptor (EGFR) and has been extensively studied for the treatment of metastatic colorectal carcinoma (CRC) [2]. Adverse effects noted with cetuximab therapy include infusion-related events in 72-90% of patients, dermatological manifestations in 30-90% of patients, gastrointestinal complaints in 19-59% of the patient population, and headaches in less than 19% of patients. Pulmonary complications with cetuximab as anticancer therapy in CRC have rarely been reported. We report a case of severe cetuximab-induced pneumonitis in a patient with CRC, a month after treatment with cetuximab [3].

## Case presentation

A 78-year-old Spanish male with a past medical history significant for diabetes mellitus, hypertension, and colorectal cancer presented to the ED with complaints of shortness of breath, epistaxis, and weakness for the past two weeks. The patient stated that he feels short of breath both on exertion and at rest. He also complained of a non-productive, non-bloody cough for the same duration. He reported a weight loss of 30 pounds in the past few weeks. He denied any fever, chills, chest pain, palpitations, nausea, or vomiting. He has no history of heart disease and denied features of orthopnea or dyspnea. Review of systems was otherwise unremarkable. He received two doses of the BNT162b2 mRNA vaccine, with the second dose administered four months ago. He did not receive a booster dose.

The patient was diagnosed with invasive rectal adenocarcinoma in 2018 by histopathology. He received neoadjuvant chemotherapy with capecitabine and radiation followed by low anterior resection and ileostomy reversal in early 2019. The patient developed metastatic disease in 2021 with an elevated carcinoembryonic antigen (CEA) level. A PET scan done in September 2021 showed hypermetabolic metastatic lymph nodes in the right iliac and left para-aortic regions, with bilateral lung nodules and mediastinal and hilar lymphadenopathy. The tumor was KRAS wild type and human epidermal growth factor receptor 2 (HER2) positive. He was started on a regimen of 5-fluorouracil, leucovorin calcium, and irinotecan in December 2021. In addition to the chemotherapy regimen, the patient was started on immunotherapy with cetuximab about a month prior to presentation.

Upon admission, the patient had a temperature of 98.7°F, blood pressure of 136/69 mmHg, a pulse of 84 beats per minute (bpm), and a respiratory rate (RR) of 21 breaths per minute, saturating 94% on room air (RA). Physical examination did not show any engorged neck veins. Bilateral rales were noted with increased respiratory effort on respiratory examination. The rest of the physical examination was within normal limits. The laboratory parameters were unremarkable as shown in Table 1. The CEA level was trending down. The trend of the CEA is shown in Table 2.

**Table 1:** Laboratory parameters on admission.

Laboratory Parameters	Values	Reference Range
Sodium	139	136-145 mmol/L
Potassium	4.7	3.5-5.3 mmol/L
Chloride	110	98-110 mmol/L
Blood Urea Nitrogen (BUN)	19	6-24 mg/dL
Creatinine	0.8	0.6-1.2 mg/dL
Aspartate Transaminase (AST)	31	10-36 U/L
Alanine Transaminase (ALT)	17	9-46 U/L
WBC	6.9	4.4-11 x10*3/uL
Hemoglobin	10.7	13.5-17.5 g/dL
Platelets	224	150-450 x10*3/uL

**Table 2:** CEA levels. CEA: Carcinoembryonic antigen.

	Reference value	Start of chemotherapy	During chemotherapy	At the time of admission
CEA	0.0-3.0 ng/ml	113.1	103.4	40.0

**Figure 1:**
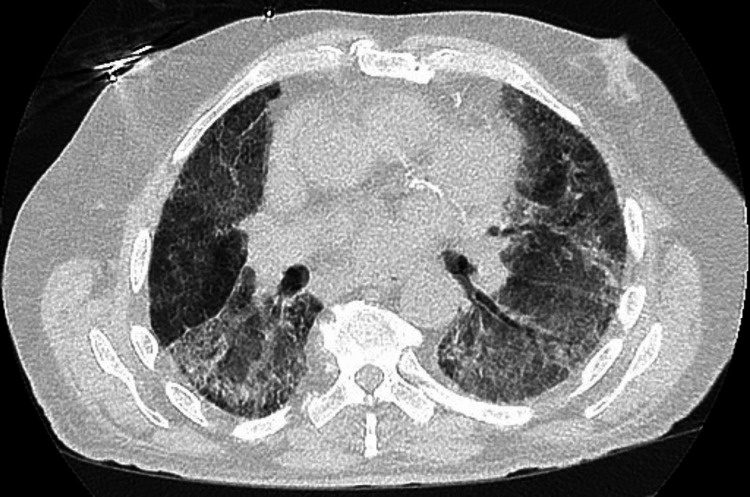
CT scan on admission showing multifocal multilobar ground-glass opacities.

**Figure 2:**
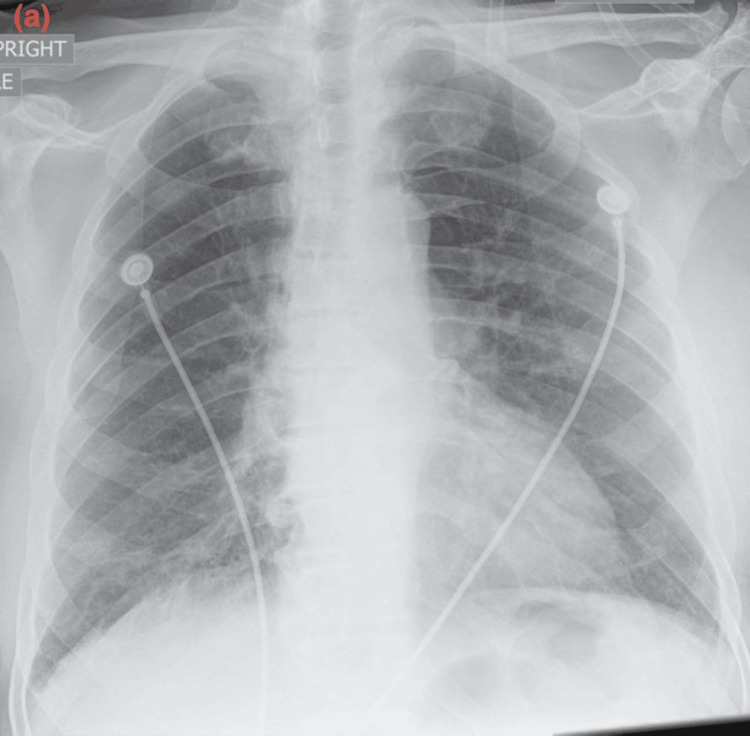
Progression of the disease on chest X-ray: (a) Chest X-ray before starting therapy showing no evidence of acute disease.

**Figure 3:**
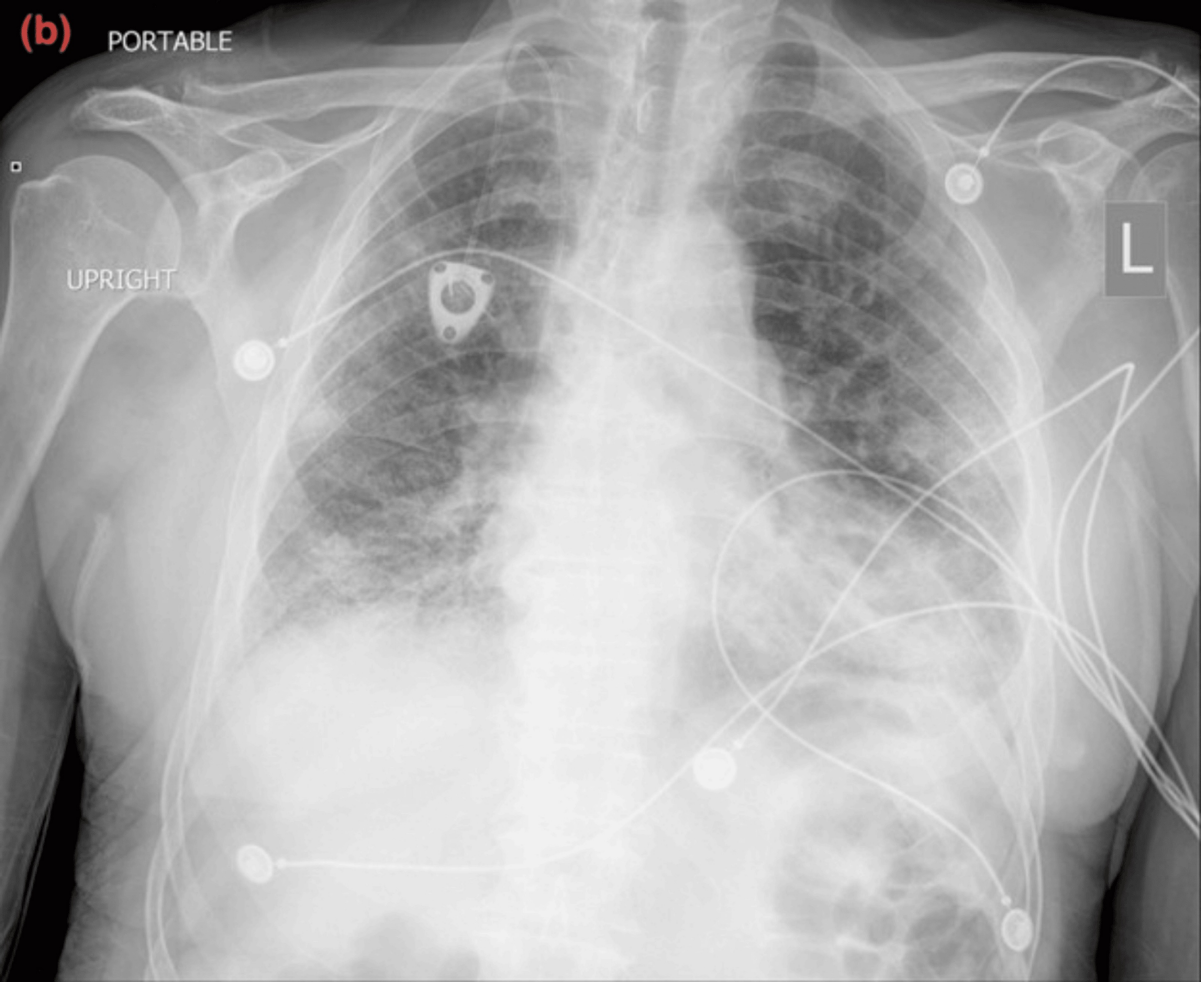
Progression of the disease on chest X-ray: (b) Chest X-ray on day of admission showing bilateral interstitial markings with patchy infiltrates.

**Figure 4:**
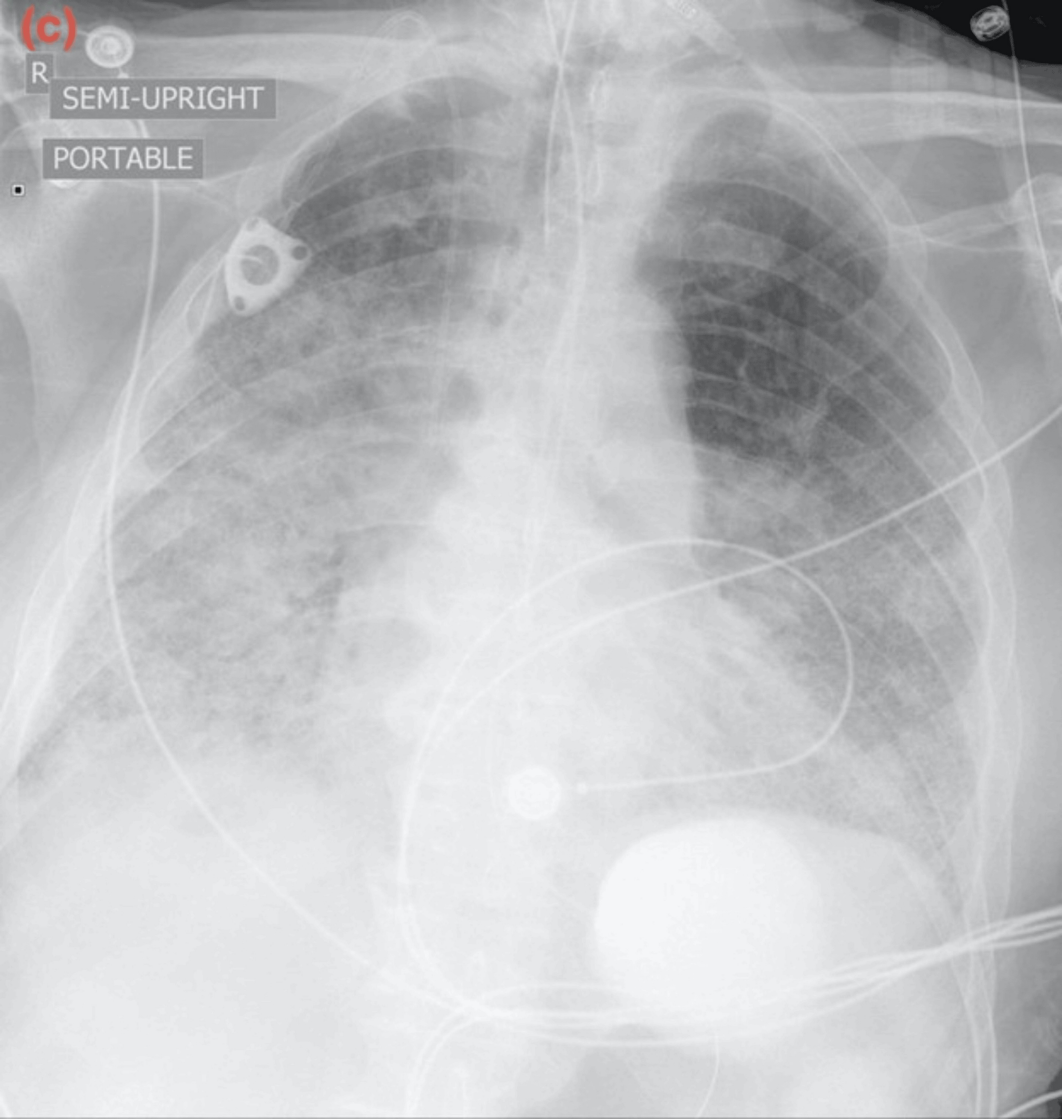
Progression of the disease on chest X-ray: (c) Chest X-ray after two weeks of hospital admission showing worsening of the bilateral infiltrates.

The chest X-ray showed bilateral interstitial markings with patchy bilateral infiltrates. The contrast tomography angiography (CTA) with IV contrast showed multifocal multilobar bilateral ground-glass opacities (as shown in Figure 1), mediastinal and right hilar lymphadenopathy, multiple lung nodules bilaterally measuring up to 5 to 6 mm, and a bilobar hepatic enhancing lesion measuring up to 13 mm. No pulmonary embolism, pneumothorax, or pleural effusion was noted. The CT scan of the sinus and facial bones with contrast was performed due to epistaxis and showed moderate left superior frontal edema with a 5 mm enhancing nodule. The CT scan of the head without contrast showed four enhancing lesions within the left frontal lobe measuring up to 6 mm in size. The electrocardiogram showed sinus rhythm without any ST-T wave changes. The echocardiography showed a left ventricular ejection fraction of 50-55% with moderate concentric left ventricular hypertrophy. The COVID-19 antigen and reverse transcriptase polymerase chain reaction (RT-PCR) tests were negative. The patient was given trimethoprim-sulfamethoxazole and cefepime for a suspected infectious etiology in an immunocompromised state. The influenza A and B RT-PCR tests were negative. The histoplasma antibody/antigen, Legionella antigen, Strongyloides IgG antibody, and cryptococcal antigen were negative. The serum Fungitell was within normal limits. The QuantiFERON Gold Plus test was indeterminate. The HIV antibody was negative. The sputum culture was normal. Bronchoscopy could not be performed due to increased oxygen requirements. During the course of the hospital stay, his oxygen requirements continued to increase, and he was transferred to the intensive care unit for worsening hypoxemia. He was placed on a high-flow nasal cannula with 50L oxygen with FiO2 70%. He was started on intravenous methylprednisolone 40 mg every 6 hours for respiratory failure, with improvement in oxygen requirement for one day. Palliative whole-brain radiation therapy was planned for the brain lesions; however, the patient was unable to tolerate it due to worsening respiratory status. The repeat chest X-ray showed worsening of the multifocal airspace disease. The trends of the chest X-ray throughout the course of the disease are shown in Figures 2-4. The patient’s oxygen requirements continued to increase despite high-dose steroid therapy, and he was intubated. He continued to deteriorate clinically and expired after two weeks of hospital admission.

## Discussion

Colorectal carcinoma is the second most common cancer in the United States [4], with 52,980 estimated deaths in 2021. Colorectal carcinoma can present as metastatic disease in approximately 25% of the patient population [5], with a 5-year survival rate between 13.8 and 14.7% for stage 4 CRC patients [6]. Standard first-line therapy, including fluorouracil with leucovorin and irinotecan or oxaliplatin, has improved the median OS to 18 months in the last decade [7]. The combination of chemotherapy with biological agents like cetuximab, bevacizumab, and panitumumab has been shown to increase the OS to more than 24 months [8].

The immunoglobulin G1 monoclonal antibody against the EGFR, cetuximab (Erbitux), is associated with improved overall survival and progression-free survival in patients with metastatic colorectal cancer (mCRC) [9]. Cetuximab is also used in non-small cell lung cancer (NSCLC) and squamous cell carcinoma of the head and neck cancer [10]. Commonly reported adverse effects of cetuximab include infusion-related reactions, fatigue, malaise, headache, and nausea [11]. Severe pulmonary complications after anticancer therapy with cetuximab have rarely been reported. We report a case of drug-induced lung injury (DLI) in a patient on cetuximab for mCRC.

DLI, including interstitial pneumonitis, is a serious adverse effect that may result in respiratory failure and death. It can be due to a direct cytotoxic effect on the lung tissues, or an indirect effect in which the lung tissues are affected by the host’s inflammatory and immunological response [12]. Interstitial pneumonitis is an umbrella term for a broad range of disorders of lung parenchyma that have similar radiological and physiological manifestations [13]. Clinically, pneumonitis is defined as a syndrome with predominant features of shortness of breath, hypoxia, and fever accompanied by pulmonary infiltrates on chest X-ray or CT scan [14]. The diagnosis of interstitial pneumonitis is based on pathological, clinical, and radiographic features, as there is no single affirmative diagnostic test [15].

In our case, the diagnosis of cetuximab-induced interstitial pneumonitis was made by the preceding use of cetuximab, lack of response to antibiotics, imaging consistent with ground-glass opacity, initial positive response to high-dose steroids, an immunocompromised state without a booster dose during the time of the rise of the Omicron variant, and the exclusion of other possible causes of pneumonitis. The diagnosis of COVID-19 was initially considered due to the imaging features; however, the COVID RT-PCR and antigen tests were negative twice. The timing of the pneumonitis was also compatible with drug-induced pneumonitis, since the median time of onset of cetuximab-related pneumonitis is 101 days after treatment initiation [12]. Our patient developed symptoms one month after treatment with cetuximab therapy.

Although cases of cetuximab-induced pneumonitis have been reported in patients of Asian descent, a similar occurrence has not commonly been described in the Spanish population. A study conducted by Hoag JB et al. showed that the overall incidence of pulmonary complications after treatment with cetuximab in patients with CRC in Japan was 3.41%, whereas the incidence of pulmonary adverse effects was 18.7% in patients with NSCLC [2]. Another study conducted by Satoh T et al. showed that the incidence of lung injury after cetuximab therapy was significantly higher in patients with prior lung disease [12]. Jain A et al. concluded in their study that pneumonitis after cetuximab was associated with a more fulminant course [1] and required aggressive management.

Management of drug-induced pneumonitis typically involves discontinuing the offending agent and initiating supportive care measures, such as supplemental oxygen and high-dose intravenous corticosteroids [1]. Our patient showed an initial response to steroid therapy; however, his condition worsened progressively despite the suggested therapy. Although no particular trend for the time of onset of lung injury has been identified with a worse outcome, lung injury within 90 days of treatment initiation has been shown to have a poor prognosis [12]. Therefore, close monitoring of clinical symptoms with early initiation of diagnostic modalities like high-resolution CT is imperative for the prompt diagnosis and treatment.

## Conclusions

This case alerts clinicians to the unique occurrence of drug-induced pneumonitis following cetuximab therapy and serves as a principal reference to distinguish cetuximab-associated pneumonitis from other infectious etiologies, including COVID-19. It also highlights the need for early detection and effective treatment of cetuximab-induced pulmonary complications.
